# Improved Sensitivity of Quantitative Polymerase Chain Reaction and Next Generation Sequencing for Detection of *Salmonella* spp. in Mixed Environmental Communities Using Whole Genome Amplification

**DOI:** 10.1002/mbo3.70194

**Published:** 2025-12-12

**Authors:** Ann Arfken, Jeffrey Mercante, Mia Mattioli

**Affiliations:** ^1^ Division of Foodborne, Waterborne and Environmental Diseases National Center for Emerging and Zoonotic Infectious Diseases, CDC Atlanta Georgia USA

**Keywords:** metagenomics, multiple displacement amplification, whole genome amplification

## Abstract

Detecting pathogens in environmental samples using molecular‐based technologies can be challenging, particularly in low biomass environments or where pathogens represent a low percentage of the community. Multiple displacement amplification (MDA) is a whole genome amplification (WGA) method that has been developed for low biomass samples. However, there is a lack of information on how MDA could improve PCR and sequence‐based detection and genomic characterization of pathogens in challenging environmental samples. In this study, MDA was evaluated on low template samples of the *Salmonella* LT2 isolate, a foodborne and waterborne environmental pathogen. MDA was also evaluated on a variety of low template mixed‐microbial mock, environmental communities containing a range of *Salmonella* genome percentages to simulate different levels of *Salmonella* in the environment. Using MDA starting inputs of 1.8 × 10^4^–1.8 × 10^1^ *Salmonella* LT2 genome copies, > 99% of the *Salmonella* genome was recovered following MDA at > 16X depth of coverage from as few as 500,000 merged, 250 bp paired‐end reads. For the mock microbial communities, moderately high levels of genome abundance distortion were evident following MDA across all communities when compared to the expected compositions, which could not be attributed to either genome size or GC content alone. Overall, MDA may provide a useful method for increasing *Salmonella* detection sensitivity in low target environmental samples where downstream selective targeted applications such as real‐time PCR or targeted amplicon sequencing are used, but MDA may not be appropriate for identification and detection of *Salmonella* when using untargeted, metagenomic sequencing.

## Graphical abstract

Multiple displacement amplification (MDA) improved qPCR detection sensitivity of a waterborne pathogen (*Salmonella*) in low biomass, mixed microbial communities but resulted in underrepresentation of the *Salmonella* genome relative to the community following metagenomic sequencing. This study demonstrates that MDA may be useful for increasing detection of pathogens in targeted molecular assays but may be unreliable for evaluating pathogens using untargeted, metagenomic sequencing approaches.

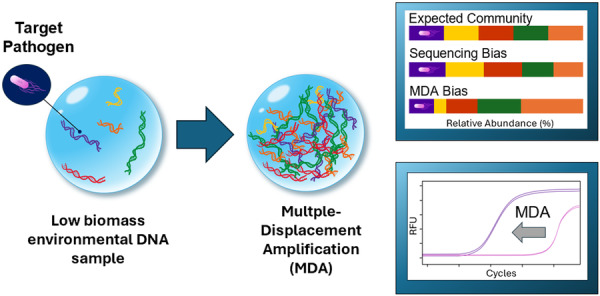

## 1 Introduction

Water‐transmitted pathogen detection and genomic characterization in environmental samples is important for monitoring exposure risk and responding to outbreaks (Andrews et al. 2020; Matrajt et al. 2020; Ramírez‐Castillo et al. 2015). However, water‐transmitted pathogens are often difficult to isolate from the background microbial communities in environmental samples, and even if feasible, pathogen culture methods are time consuming, some taking as many as 10 days. Moreover, many pathogens, such as viruses and parasites, do not have laboratory culture methods, or prove difficult to culture, such as *Salmonella* Typhi (Cho and Kim 1999; Karkey et al. 2016). Therefore, to decrease outbreak response time and better understand water‐transmitted pathogen risks from the environment, culture‐independent methods have emerged, such as metagenomics and targeted sequencing methods, to detect and type a variety of pathogens (Carleton et al. 2019; F. Li et al. 2021; Sato et al. 2019).

Despite the increased focus on developing culture‐independent environmental pathogen detection methods, genetically linking pathogens in the environment to outbreak‐associated clinical cases is still challenging. This is because oftentimes pathogens exist at low concentrations in the environment and may occur with a relatively high concentration of nontarget, sometimes closely genetically related, microorganisms (Bass et al. 2023). For example, drinking water is an environment where both pathogens and other nontarget microorganisms often coexist in low concentrations (Jofre and Blanch 2010), whereas surface water may be abundant in nontarget microorganisms but low in relative pathogen levels (Straub and Chandler 2003).

One potential strategy for overcoming the low biomass or limited DNA target hurdle in environmental samples is through whole genome amplification (WGA). WGA methods amplify DNA in a sequence‐independent manner by using random or partially random primers, supporting a variety of downstream applications including microarrays and metagenomic sequencing (Volozonoka et al. 2022). Multiple displacement amplification (MDA) is one such WGA method that has been used in low biomass samples to amplify DNA (Ahsanuddin et al. 2017; Handyside et al. 2010; Wu et al. 2006). Compared to other WGA methods such as multiple annealing and looping‐based amplification (MALBAC) and degenerate oligonucleotide‐primed PCR (DOP‐PCR), MDA is often simpler to implement, available as a commercial kit, and utilizes Phi29 DNA polymerase (Φ29), which allows for higher processivity and fidelity (J. Li et al. 2017).

Several studies have used MDA to amplify DNA in environmental samples for metagenomic community analyses applications (Abulencia et al. 2006; Ahsanuddin et al. 2017; Direito et al. 2014). However, WGA performance for pathogen detection in environmental communities using MDA remains unknown. In this study, we evaluated the ability of MDA to increase pathogen detection sensitivity as determined by quantitative polymerase chain reaction (qPCR) and next generation sequencing in mock environmental water samples, as well as the impact of the background microbial community. The water‐transmitted pathogen, *Salmonella*, was used as the target environmental pathogen for the evaluation.

## 2 Methods

### 2.1 Isolate and Mock Communities

Purified *Salmonella enterica* subsp. *enterica* serovar Typhimurium str. LT2 (ATCC 19585) DNA and two synthetic environmental, mock DNA communities, ZymoBIOMICS Microbial Community Standard (catalog no. D6305, Zymo) and ATCC ABRF‐MGRG 10 strain even mix (catalog no. MSA‐3001, ATCC), were used to simulate pathogens in the environment for MDA evaluation. *S. enterica* subsp. *enterica* strain FDAARGOS 609 (GCF_006370495.1) is included in the Zymo commercial DNA standard, while the ATCC environmental mock community did not initially contain any *Salmonella* spp. These mock communities provided standardized and quantified material for comparison across experiments, while still representing the diversity of taxa often present in environmental water where the target or organism, *Salmonella*, can be found. Further details regarding the commercial mix compositions are provided in Table A1.

Purified *Salmonella* LT2 DNA was serially diluted with 0.1X Tris‐EDTA (TE) buffer in duplicate sample series (A and B) as follows: 1.8 × 104 (0.1 ng), 1.8 × 10^3^ (0.01 ng), 1.8 × 10^2^ (0.001 ng) and 1.8 × 10^1^ (0.0001 ng) estimated genome copy numbers. These samples are henceforth referred to as “isolate samples.” The Zymo commercial mix was also serially diluted ten‐fold in duplicate (samples series A and B) as follows: 1, 0.1, 0.01, and 0.001 ng, which corresponded to 2.3 × 10^4^, 2.3 × 10^3^, and 2.3 × 10^2^ estimated *Salmonella* FDAARGOS 609 genome copies, respectively. Finally, purified *Salmonella* LT2 DNA was spiked at dilutions of 1.8 × 10^1^ (0.0001 ng), 1.8 × 10^2^ (0.001 ng) and 1.8 × 10^3^ (0.01 ng) estimated genome copies into 1, 0.1, 0.01, and 0.001 ng each (12 samples total, no duplicates) of the ATCC environmental community to simulate various ratios of a target pathogen in a background microbial community.

The list of samples tested for the MDA evaluation is provided in Table 1, including the target pathogen estimated genome copies and total DNA in each sample. Sample names for the isolate and Zymo communities correspond to the log10 order of the *Salmonella* genome copy number and the percent of *Salmonella* in the total MDA community inputs (e.g., 10^4^−10 corresponds to 10^4^ genome copies and 10% *Salmonella*). The sample names for the ATCC environmental community follow a similar naming scheme but also include the initial mass (ng DNA) of the ATCC environmental community before being spiked with the *Salmonella* isolate (e.g., 10^2^_0.1_(1) corresponds to 10^2^ genome copies, 0.1% Salmonella, and 1.0 ng of community input).

**Table 1:** Composition of isolate and mock community templates for MDA analysis

Sample name^a^	Sample type	*Salmonella* strain	Replicate	*Salmonella* (genome copy number)	*Salmonella* input (ng DNA)	Community input (ng DNA)	*Salmonella* (%) community
10^4^_100‐A	Isolate	LT2	A	18,000	0.1	0	100.0
10^4^_100‐B	Isolate	LT2	B	18,000	0.1	0	100.0
10^3^_100‐A	Isolate	LT2	A	1800	0.01	0	100.0
10^3^_100‐B	Isolate	LT2	B	1800	0.01	0	100.0
10^2^_100‐A	Isolate	LT2	A	180	0.001	0	100.0
10^2^_100‐B	Isolate	LT2	B	180	0.001	0	100.0
10^1^_100‐A	Isolate	LT2	A	18	0.0001	0	100.0
10^1^_100‐B	Isolate	LT2	B	18	0.0001	0	100.0
10^4^_10‐A	Zymo	FDAARGOS 609	A	23,000	0.1	0.9	10.0
10^4^_10‐B	Zymo	FDAARGOS 609	B	23,000	0.1	0.9	10.0
10^3^‐10‐A	Zymo	FDAARGOS 609	A	2300	0.01	0.09	10.0
10^3^_10‐B	Zymo	FDAARGOS 609	B	2300	0.01	0.09	10.0
10^2^_10‐A	Zymo	FDAARGOS 609	A	230	0.001	0.009	10.0
10^2^_10‐B	Zymo	FDAARGOS 609	B	230	0.001	0.009	10.0
10^1^_10‐A	Zymo	FDAARGOS 609	A	23	0.0001	0.0009	10.0
10^1^_10‐B	Zymo	FDAARGOS 609	B	23	0.0001	0.0009	10.0
10^3^_1.0_(1)	ATCC environmental	LT2	NA	1800	0.01	1	1.0
10^2^_0.1_(1)	ATCC environmental	LT2	NA	180	0.001	1	0.1
10^1^_0.01_(1)	ATCC environmental	LT2	NA	18	0.0001	1	0.0
10^3^_9.1_(0.1)	ATCC environmental	LT2	NA	1800	0.01	0.1	9.1
10^2^_1.0_(0.1)	ATCC environmental	LT2	NA	180	0.001	0.1	1.0
10^1^_0.1_(0.1)	ATCC environmental	LT2	NA	18	0.0001	0.1	0.1
10^3^_50.0_(0.01)	ATCC environmental	LT2	NA	1800	0.01	0.01	50.0
10^2^_9.1_(0.01)	ATCC environmental	LT2	NA	180	0.001	0.01	9.1
10^1^_1.0_(0.01)	ATCC environmental	LT2	NA	18	0.0001	0.01	1.0
10^3^_90.9_(0.001)	ATCC environmental	LT2	NA	1800	0.01	0.001	90.9
10^2^_50.0_(0.001)	ATCC environmental	LT2	NA	180	0.001	0.001	50.0
10^1^_9.1_(0.001)	ATCC environmental	LT2	NA	18	0.0001	0.001	9.1

*Note:* Genome copy numbers were estimated based on the amount of ng DNA *Salmonella* in the sample template and the appropriate *Salmonella* spp. genome size. Community ng DNA input includes all other microbial DNA, excluding *Salmonella* spp.

^a^ Sample names for the Isolate and Zymo communities correspond to the log10 order of the *Salmonella* genome copy number and the percent of *Salmonella* in the total MDA community inputs; A & B indicate duplicates. Sample names for the ATCC environmental correspond to the log10 order of the *Salmonella* genome copy number, the percent of *Salmonella* in the total MDA community, and the initial mass (ng DNA) input of the ATCC environmental community before being spiked with the *Salmonella* isolate.

### 2.2 WGA

MDA was performed on the isolate and mock community samples using the REPLI‐g Single Cell WGA kit (catalog no. 150345, Qiagen) following the purified gDNA protocol. During MDA, samples were incubated at 30°C for the manufacturer recommended 16 h to achieve maximum DNA yield. Duplicate no template control (NTC) and positive template controls were included in each MDA reaction. Positive controls consisted of 10 ng of human genomic DNA (catalog no. D7192, Sigma Aldrich) for the isolate samples and 10 ng of *Salmonella* LT2 genomic DNA for the mock communities (Zymo and ATCC). NTCs consisted of 2 µL of molecular grade water. To evaluate the impact of sequencing bias, an aliquot of isolate DNA or mock community DNA (non‐diluted, non‐spiked) without undergoing MDA was sequenced alongside the MDA samples to serve as the non‐MDA sequencing controls. DNA concentrations were measured using Qubit Fluorometer Quantification (Invitrogen, Waltham, MA), and DNA quality was assessed for size and DNA integrity number (DIN) with a TapeStation 2200 (Agilent, Santa Clara, CA).

### 2.3 qPCR Assay

qPCR was used to quantify the increase in *Salmonella* genomes, as represented by *ttr* gene quantity, following MDA. Previously described primers (Forward: 5′‐CTCACCAGGAGATTCAACATGG‐3′, Reverse: 5‐AGCTCAGACCAAAAGTGACCATC‐3′) and TaqMan probe (5′‐Fam‐CACCGACGGCGAGACCGACTTT‐BHQ1‐3′) were used to amplify the highly conserved single‐copy *Salmonella* spp. *ttr* gene (Malorny et al. 2004) before and after MDA. Each qPCR reaction was performed in duplicate using a 20‐µL reaction volume containing 10 µL of 1X ABI Environmental Master Mix 2.0 (catalog no. 4396838, Life Technologies), 0.4 µM of both forward and reverse primers, 0.25 µM FAM‐labeled probe, and TaqMan 1X VIC‐labeled exogenous DNA internal amplification control (catalog no. 4308321, ThermoFisher). Each 96‐well plate was run with duplicate qPCR no template controls and a six‐point standard curve of serially diluted *Salmonella* LT2 isolate DNA starting at 3.68 × 10^5^ genome copies. Quantitative PCR assays were conducted on an ABI 7500 Real‐Time PCR System with the following cycling parameters: an initial denaturation at 95°C for 10 min followed by 45 cycles of denaturation at 95°C for 15 s, then annealing and fluorescence acquisition at 60°C for 1 min. Quantitative results were averaged from sample duplicates.

### 2.4 Sequencing

Sequencing libraries were prepared using the NEBNext Ultra II FS Kit (catalog no. E7805L, New England Biolabs), following the protocol for DNA inputs ≥ 100 ng (100–500 ng range) and a fragmentation incubation time of 15 min. To account for potential branching and amplification artifacts from MDA, 500 ng of MDA sample DNA was used as starting inputs for library prep, while 100 ng of genomic DNA was used as starting inputs for library prep with non‐MDA sequencing control samples. This resulted in similar final library concentrations of both MDA and non‐MDA samples. Sequencing blanks consisting of nuclease free water were included for each library preparation. Library quantification was performed with the KAPA Library Quantification Kit (catalog no. KK4824, Roche) on an ABI 7500 following manufacturer's instructions. Normalized, pooled libraries for each community were sequenced on the Illumina MiSeq Sequencing platform with the MiSeq Reagent V2 Kit (catalog no. MS‐102‐2003, Illumina) using a 2 × 250 bp (base pair) sequencing protocol.

### 2.5 Bioinformatics

#### 2.5.1 Quality Control

Raw paired‐end fastq reads were trimmed for quality, length, and adapter removal with Fastp (version 0.20.1)(Chen et al. 2018) using the following parameters: ‐r –cut_right_window_size 4 –cut_right_mean_quality 15 and –length_required 50. Paired‐end reads were merged using BBMap (version 38.90) (https://sourceforge.net/projects/bbmap/). High quality reads were defined as adapter‐free, merged paired‐end reads with lengths > 50 bp and minimum mean quality scores > 15 averaged over four consecutive bps.

#### 2.5.2 Assigning Taxonomic Composition

High quality reads from the MDA isolate or mixed mock community samples and the non‐MDA sequencing controls were multi‐mapped to individual reference genomes using customized reference databases and default parameters with FastQ‐Screen (version 0.15.2) (Wingett and Andrews 2018), a metagenomic, mapping‐based tool. For the isolate samples, the reference database consisted of: NCBI reference genome *Salmonella* LT2 (GCF_000006945.2), common laboratory contaminants *Escherichia coli* (GCF_000004845.2) and human (GCF_000001405.39), and reagent contaminants PhiX (NC_001422), Φ29 (NC_011048.1) and vectors (http://www.ncbi.nlm.nih.gov/VecScreen/UniVec.html). For the commercial mock community analyses, PhiX, Φ29, vectors and the *Salmonella* LT2 genome (where appropriate) were included in the reference databases, as well as reference genomes specific to each community based on the manufacturer's instructions. Reads assigned to multiple genomes from FastQ‐Screen were excluded from taxa relative abundance comparisons between the theoretical expected percentages and the non‐MDA sequencing controls. However, relative abundance comparisons between non‐MDA sequencing controls and the MDA experimental samples did include multiple genome hits to allow for direct comparisons.

Reads that multi‐mapped to multiple genomes were extracted using the filtering commands with FastQ‐Screen. A BLAST search was conducted using the extracted reads against the mock communities' respective reference databases with NCBI‐blast+ (Camacho et al. 2009). Thresholds for returned hits were set at a minimum percent identity of 90%, a minimum query coverage of 90%, 1 alignment per query‐subject pair, and 5 maximum returned top hits (blast flags: ‐perc_identity 90 ‐qcov_hsp_perc 90 ‐max_hsps 1 ‐max_target_seqs. 5). Top hits for each query were based on the highest bit score. Untied top hits were assigned to the appropriate single taxon and tied hits were assigned to the appropriate multiple taxa. Since samples were comprised of known isolates or defined mock communities, thresholds were set to allow for some error introduced during sequencing (Stoler and Nekrutenko 2021).

#### 2.5.3 Impact of MDA on *Salmonella* spp. Sequencing Performance

*Salmonella* sequencing performance was evaluated for genome breadth of coverage, mean read depth, and coverage uniformity (coefficient of variation; CV) across the different experiments (Table 1) and at multiple sequencing depths. For each sequencing depth, total merged paired‐end sample reads were individually mapped (single‐mapping approach) to the appropriate *Salmonella* reference genome, LT2 or FDAARGOS 609, with Bowtie2 (version 2.4.5) (Langmead and Salzberg 2012) using a strict mapping threshold with the following function *f*(*x*) = 0 + −0.2 * x defining the minimum score alignment for each read (bowtie2 flag: ‐‐score‐min L,0,−0.2). This allowed for the exclusion of poorly mapped or ambiguous reads and reduced the likelihood of reads that potentially map to more than one genome. Subsampling was conducted using the SeqKit (version 2.4.0) (Shen et al. 2016). The *Salmonella* LT2 plasmid was excluded from the performance statistics. The NCBI *Salmonella* FDAARGOS 609 reference genome does not contain a plasmid.

Samtools (version1.13) (Danecek et al. 2021) was used to determine breadth of coverage and mean read depth of reads mapped to the appropriate *Salmonella* genome. Coverage uniformity was calculated by dividing the sample standard deviation of coverage depth per reference genome position by the mean coverage depth. Genome coverage comparisons between the different experiments were assessed at a sequencing depth of 742,454 reads, which corresponds to the lowest sample sequencing depth within one standard deviation from the mean number of MDA sample reads, which allowed for the greatest number of samples to be compared without compromising read depth. The mean read depth for the *ttr* gene was calculated for positions 1,469,333–1,469,427 bp and 3,981,266–3,981,360 bp on the LT2 and FDAARGOS reference genomes, respectively. These positions correspond with the amplified *ttr* gene fragment from the qPCR assay. GC bias in the MDA isolate samples was calculated using Genome Analysis Toolkit (GATK) (version 4.1.8.0) (McKenna et al. 2010) Picard's CollectGcBiasMetrics command with a window size of 200.

#### 2.5.4 NTC and Positive Controls

Both NTC and positive controls for isolate and mock communities were multi‐mapped to the appropriate customized genome databases using Fastq‐Screen. Based on the high number of *Escherichia coli* reads present in all REPLI‐g NTC controls from the Fastq‐Screen results, reads from the negative controls were mapped separately to the *E. coli* reference genome using the strict Bowtie2 mapping criteria to assess mapping performance and depth profiles, which are not available with the multi‐genome mapping Fastq‐Screen tool. Control mapping results are provided in the Appendices under “Control Composition” (Figures A1 and A2, Tables A2 and A3).

#### 2.5.5 Data Visualizations

Relative abundances of mapped reads and genomic performance metrics (breadth of coverage, mean read depth, and CV) were visualized in R (version 3.6.1) (https://www.r‐project.org/) using ggplot2 (version 3.3.6) (Wickham 2016). Results from the *ttr* gene qPCR assays, genome coverage plots, and isolate GC bias curves were visualized in Python (version 3.9.13) (https://www.python.org/) using Matplotlib (version 3.5.3). Mean *ttr* gene counts for duplicate samples were plotted for the isolate and Zymo community samples. Coverage plots were based on the minimum sample sequencing depth, as mentioned above. For isolate coverage and GC bias plots, the LT2 non‐MDA sequencing control and MDA positive control (starting input of 10 ng DNA of *Salmonella* LT2) samples were included for comparison. The non‐MDA sequencing control for the Zymo community was also included in the Zymo coverage plots. For communities with duplicate samples, coverage plots (isolate and Zymo) and GC bias plots (isolate DNA) for only one sample was selected for visualization in the main text. The duplicate coverage plots are provided in the Appendices (Figures A3 and A4).

### 2.6 Statistical Analysis

Spearman rank correlation analyses using R were conducted to assess the relationships between starting input DNA amounts and MDA output DNA concentrations. Pearson correlation analyses in R were used to determine the relationships between and the relative abundance differences (differences between expected commercial abundances and non‐MDA sequencing controls) and the (1) log10 transformed genome size or (2) genome %GC content. Normality of data was confirmed before performing Pearson correlation analyses was using Shapiro‐Wilk's test and visualized with qqplots in R. All errors are given as standard error unless otherwise indicated, and significance was determined as *p* < 0.05.

## 3 Results

### 3.1 MDS

Following MDA, isolate and mock community samples had a mean output of 1.1 × 10^3^ ± 1.0 × 10^2^ ng/µL DNA. The MDA DNA yield was consistent among samples regardless of starting input (0.001–1.01 ng DNA) with no significant correlation between starting DNA inputs and output yield following MDA for any sample (isolate: *r*(6) = 0.08, *p* = 0.86; Zymo: *r*(8) = 0.10, *p* = 0.81; ATCC: *r*(8) = 0.05, *p* = 0.87). The fold change between the starting mass of DNA and final DNA outputs ranged from 2.9 × 10^4^–9.8 × 10^8^‐fold increase (Table 2). No template REPLI‐g controls outputs ranged from 4.2 × 10^4^–7.0 × 10^4^ ng DNA, and positive template control outputs with starting DNA inputs of 10 ng ranged from 3.0 × 10^4^–1.3 × 10^5^ ng DNA (Table A4). Of note, approximately 40 µg of DNA is expected in negative controls following MDA per the REPLI‐g manufacturer's protocol due to random extension of primer dimers.

**Table 2:** Starting input DNA amounts and final output DNA concentration and amounts following WGA with REPLI‐g MDA for all samples.

Sample name	Description	MDA input total DNA (ng)	MDA output concentration Qubit (ng/µL)	MDA output total DNA (ng)^a^	Fold change (X)
10^4^_100‐A	*Salmonella* isolate MDA	0.1	1.3E + 03	6.4E + 04	6.4E + 05
10^4^_100‐B	*Salmonella* isolate MDA	0.1	1.2E + 03	6.1E + 04	6.1E + 05
10^3^_100‐A	*Salmonella* isolate MDA	0.01	1.0E + 03	5.2E + 04	5.2E + 06
10^3^_100‐B	*Salmonella* isolate MDA	0.01	1.1E + 03	5.4E + 04	5.4E + 06
10^2^_100‐A	*Salmonella* isolate MDA	0.001	1.4E + 03	7.1E + 04	7.1E + 07
10^2^_100‐B	*Salmonella* isolate MDA	0.001	1.0E + 03	5.1E + 04	5.1E + 07
10^1^_100‐A	*Salmonella* isolate MDA	0.0001	2.0E + 03	9.8E + 04	9.8E + 08
10^1^_100‐B	*Salmonella* isolate MDA	0.0001	1.1E + 03	5.7E + 04	5.7E + 08
10^4^_10‐A	Zymo MDA	1.0	5.8E + 02	2.9E + 04	2.9E + 04
10^4^_10‐B	Zymo MDA	1.0	7.4E + 02	3.7E + 04	3.7E + 04
10^3^‐10‐A	Zymo MDA	0.1	6.3E + 02	3.2E + 04	3.2E + 05
10^3^_10‐B	Zymo MDA	0.1	7.3E + 02	3.6E + 04	3.6E + 05
10^2^_10‐A	Zymo MDA	0.01	6.9E + 02	3.5E + 04	3.5E + 06
10^2^_10‐B	Zymo MDA	0.01	6.6E + 02	3.3E + 04	3.3E + 06
10^1^_10‐A	Zymo MDA	0.001	6.5E + 02	3.2E + 04	3.2E + 07
10^1^_10‐B	Zymo MDA	0.001	6.4E + 02	3.2E + 04	3.2E + 07
10^3^_1.0_(1)	ATCC environmental MDA	1.01	2.1E + 03	1.1E + 05	1.0E + 05
10^2^_0.1_(1)	ATCC environmental MDA	1.001	6.1E + 02	3.1E + 04	3.1E + 04
10^1^_0.01_(1)	ATCC environmental MDA	1.0001	3.0E + 03	1.5E + 05	1.5E + 05
10^3^_9.1_(0.1)	ATCC environmental MDA	1.01	7.1E + 02	3.5E + 04	3.5E + 04
10^2^_1.0_(0.1)	ATCC environmental MDA	1.001	7.1E + 02	3.5E + 04	3.5E + 04
10^1^_0.1_(0.1)	ATCC environmental MDA	1.0001	1.0E + 03	5.2E + 04	5.1E + 04
10^3^_50.0_(0.01)	ATCC environmental MDA	1.01	1.1E + 03	5.4E + 04	5.3E + 04
10^2^_9.1_(0.01)	ATCC environmental MDA	1.001	1.1E + 03	5.5E + 04	5.4E + 04
10^1^_1.0_(0.01)	ATCC environmental MDA	1.0001	9.6E + 02	4.8E + 04	4.8E + 04
10^3^_90.9_(0.001)	ATCC environmental MDA	1.01	1.3E + 03	6.3E + 04	6.2E + 04
10^2^_50.0_(0.001)	ATCC environmental MDA	1.001	1.6E + 03	7.8E + 04	7.8E + 04
10^1^_9.1_(0.001)	ATCC environmental MDA	1.0001	1.5E + 03	7.6E + 04	7.5E + 04

*Note:* Sample names for the Isolate and Zymo communities correspond to the log10 order of the *Salmonella* genome copy number and the percent of *Salmonella* in the total MDA community inputs; A and B indicate duplicates. Sample names for the ATCC environmental correspond to the log10 order of the *Salmonella* genome copy number, the percent of *Salmonella* in the total MDA community, and the initial mass (ng DNA) input of the ATCC environmental community prior to before being spiked with the *Salmonella* isolate.

^a^ in 50 µL output volume.

### 3.2 *Salmonella ttr* Gene

#### 3.2.1 Quantitative PCR of TTR Gene

**Figure 1:**
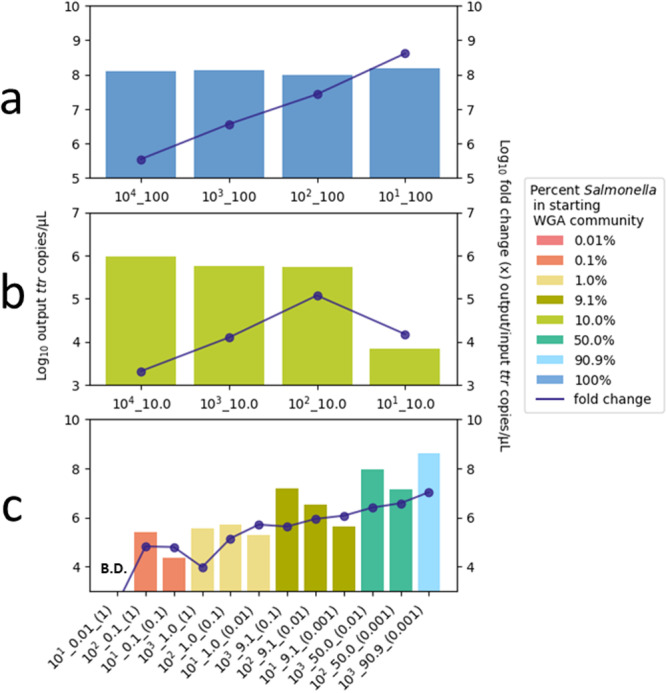
Quantification of the *ttr* gene following MDA. Quantitative PCR assay results for the *Salmonella*‐specific *ttr* gene in MDA outputs from (a) no competition, pure Isolate (100% *Salmonella*) (b) even competition, Zymo mixed‐microbial (10% *Salmonella*) and (c) varying competition, ATCC environmental mixed‐microbial (0.1%–90.6% *Salmonella*) starting input communities. Mean *ttr* copy numbers based on sample duplicates are given for (a) isolate and (b) Zymo communities. B.D. indicates below detection limit. Left *y*‐axis is the log10 output *ttr* copies/µL and right y‐axis is the log10 fold change in copies/µL between MDA input and output. Sample names for the (a) isolate and (b) Zymo community correspond to the log10 order of the *Salmonella* genome copy number and the percent of *Salmonella* in the total MDA community inputs. Sample names for the (c) ATCC environmental community correspond to the log10 order of the *Salmonella* genome copy number, the percent of *Salmonella* in the total MDA community, and the initial mass (ng DNA) input of the ATCC environmental community before being spiked with the *Salmonella* isolate.

The *Salmonella spp. ttr* gene was quantified in samples to determine the estimated abundance of *Salmonella* following MDA with the REPLI‐g kit (Figure 1, Table A5). Quantitative results were averaged for isolate and Zymo mock community duplicates. Most samples produced > 1 ng *Salmonella* DNA/µL of MDA output for *Salmonella* spp. inputs ranging from 0.0001 to 0.1 ng/µL). The exceptions to this were: (1) the Zymo community with a starting input of 0.0001 *Salmonella* ng DNA (Zymo sample: 10^1^_10), and 2) the ATCC environmental communities with starting inputs of 1 and 0.1 ng spiked with 1.8 × 10^1^ copies of the *Salmonella* genome (ATCC environmental samples: 10^1^_0.01_(1) and 10^1^_0.1_(0.1)). Fold changes in copies of the *ttr* gene ranged from a 3.5 ×10^5^–4.1 × 10^8^ increase in the isolate community to a 9.6 × 10^3^–1.1 × 10^7^‐fold increase in the mixed mock communities.

In isolate samples, MDA resulted in an estimated 8.0 log_10_ copies/µL (range: 1.1 × 10^8^–1.5 × 10^8^) of the *ttr* gene regardless of starting DNA mass input. This equated to a 3.5 × 10^5^‐fold increase in the highest starting input of Salmonella DNA (0.1 ng) and a 4.1 × 10^8^‐fold increase in the lowest starting input of *Salmonella* DNA (0.0001 ng). Zymo mock community samples showed an overall increasing trend of *Salmonella* genome MDA output with increasing concentrations of mock community DNA input. Starting inputs of 0.001 ng mock community DNA showed the greatest increase in *ttr* gene copies/µL output, which was an increase of two orders magnitude higher concentration compared to the highest input of mock community DNA.

In the ATCC environmental community samples, higher input *Salmonella* DNA to mock community DNA ratios resulted in higher output concentrations of *ttr* gene copies/µL. The *ttr* gene output amounts were highest from ATCC environmental samples spiked with the highest amount of *Salmonella* DNA (1.8 × 10^3^ genome copies). MDA outputs for the highest spiked samples increased from 9.6 × 10^5^ to 3.9 × 10^8^ *ttr* gene copies/µL in mock communities of 0.001 ng DNA to 1.0 ng community DNA, respectively. At the lowest spiked *Salmonella* inputs (1.8 × 10^1^ genome copies), MDA outputs ranged from 4.4 × 10^5^ *ttr* gene copies/µL to below the limit of detection (LOD = 0.1% *Salmonella* DNA in ≥ 0.1 ng DNA mixed community) in mock communities of 0.001 ng DNA to 1.0 ng community DNA, respectively.

#### 3.2.2 Sequencing of *ttr* Gene Region

To determine the effects of MDA on potential amplification bias of the *ttr* gene, the mean *ttr* gene read depth, as a proportion of the overall mean genome read depth from MDA sequencing, was compared in the isolate samples (Table A6). Sample analysis was limited to the isolate samples due to the generally low mean read depth and breadth of coverage of *Salmonella* spp. in the mixed‐microbial mock communities. In general, the *ttr* gene region targeted by qPCR was underrepresented in the genome sequencing data following MDA. Read depths for the *ttr* gene were between 1.5 and 6.1 times lower than genome mean read depths except for one sample with an input of 1.8 × 10^1^ ng *Salmonella* DNA, which had 1.8 times greater read depth for the *ttr* gene than the corresponding overall mean *Salmonella* genome read depth.

### 3.3 Sequencing Analysis of Mock Communities and Isolates

#### 3.3.1 Sequencing QC

A total of 32 isolate or mock community samples (Isolate: 8; Zymo: 8; ATCC: 12; non‐MDA sequencing controls: 3) were sequenced with a mean high quality, trimmed, and merged paired‐end sequencing read depth of 1.4 × 10^6^ ± 1.0 × 10^5^ reads (Table A7). Contamination was negligible in library preparation no template controls for all sequencing runs (≤ 27 reads).

#### 3.3.2 MDA Impact on Isolate Samples

**Figure 2:**
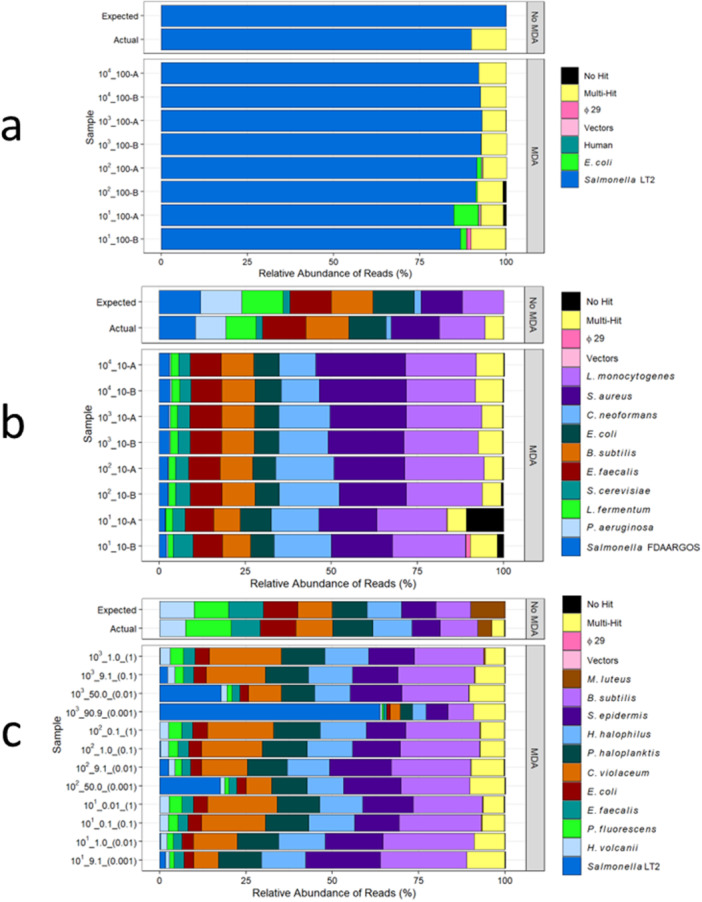
Effect of input *Salmonella* copy numbers on community composition following MDA. Taxonomic composition of (a) isolate (100% *Salmonella*) (b) Zymo (10% *Salmonella*) and (c) ATCC environmental (mixed % *Salmonella*) communities based on relative abundances of mapped reads. Reads were multi‐mapped using Fastq‐Screen and a customized database, which included the appropriate community references and common nontarget contaminants. The predicted community composition (Expected) and a non‐MDA sequencing control that did not undergo MDA (Actual) are included for comparison. Reads that did not map (No Hit) and reads that mapped to more than one reference genome (Multi‐Hit) in the database are also included. Sample names for the (a) isolate and (b) Zymo community correspond to the log10 order of the *Salmonella* genome copy number and the percent of *Salmonella* in the total MDA community inputs. Sample names for the (c) ATCC environmental community correspond to the log10 order of the *Salmonella* genome copy number, the percent of *Salmonella* in the total MDA community, and the initial mass (ng DNA) input of the ATCC environmental community before being spiked with the *Salmonella* isolate.

Using the community multi‐mapping approach, 89.8% of reads uniquely mapped (single hits or multiple hits on the same genome) to the *Salmonella* LT2 reference genome in the isolate non‐MDA sequencing control, and ≥ 84.9% of reads (range: 84.9%–93.0%) uniquely mapped to the genome reference in the isolate MDA samples (Figure 2A). Reads mapping to Φ29, vectors, and *E. coli* increased as *Salmonella* DNA mass inputs decreased in the MDA samples. Isolate samples with the lowest DNA input (0.0001 ng, 1.8 × 10^1^ *Salmonella* LT2 genome copies) displayed the highest percent relative abundance of reads mapped to *E. coli* (range: 1.7%–7.1%).

#### 3.3.3 MDA Impact on Mock Community Composition

Both mock communities showed some evidence of sequencing bias before MDA as shown in the non‐MDA sequencing control data. For the Zymo mock community, GC content was negatively correlated with differences in taxa relative abundances between expected abundances and the non‐MDA sequencing control abundances (*r*(8) = −0.88, *p* < 0.05). Differences in relative abundances per taxa ranged from −3.6% to +1.2%. For the ATCC environmental mock community, differences between expected abundances and the non‐MDA sequencing control were positively correlated with genome size (*r*(8) = 0.83, *p* < 0.05) except for the *Micrococcus luteus* genome, which had a −6.0% reduction in relative abundance compared to the non‐MDA sequencing control. The ATCC mock community difference in relative abundance per taxa ranged from −1.8% to +2.5%.

The difference between taxa relative abundances for the non‐MDA sequencing control and the MDA samples in the Zymo mock community varied widely (Figure 2B). However, when comparing among MDA samples, the relative abundances per taxa were similar (standard deviation (SD) range per taxa: 0.1%–3.3%). Taxa that were most overrepresented in relative abundance following MDA included *Staphylococcus aureus, Listeria monocytogenes*, and *Cryptococcus neoformans*, with mean relative abundance differences between MDA samples and the non‐MDA sequencing control of +7.1% ± 1.2%, +8.0% ± 0.4%, and +13.1% ± 0.9%, respectively. Taxa that were most underrepresented following MDA included *Lactobacillus fermentum*, *Salmonella* FDAARGOS 609, and *Pseudomonas aeruginosa* with mean relative abundance differences of −6.3% ± 0.1%, −7.7% ± 0.2%, and −8.5% ± 0.0%, respectively. While there was not a statistically significant correlation between percent GC and mean relative abundance differences, the three taxa with the greatest percent decrease in observed relative abundances following MDA also had the highest genome percent GC in the Zymo community (*L. fermentum* = 52.4% less than expected, *S*. FDAARGOS 609 = 52.2% less than expected, *P. aeruginosa* 66.2% less than expected).

Similar to the Zymo community, large variation was observed in taxa relative abundance between the ATCC environmental mock community MDA samples and the non‐MDA sequencing control. However, among the MDA samples, different input amounts did not produce a large effect on overall ATCC taxa relative abundances. Taxa relative abundances of MDA samples were all within two standard errors of the mean relative abundance per taxa (standard error (SE) range per taxa: 0.0%–1.5%). Between the MDA samples and non‐MDA sequencing control, *Bacillus subtilis* exhibited the greatest increase in relative abundance (absolute increase of 12.0% ± 0.6%), and *Pseudomonas fluorescens* exhibited the greatest decrease (absolute increase of 10.8% ± 0.3%). The taxa with the greatest percent decrease from expected relative abundances following MDA was *Micrococcus luteus* (mean absolute decrease of 11.8% ± 0.0%), which is equivalent to a 99.98% reduction in total genomic abundance in the taxa following MDA.

Overall, *Salmonella* was consistently underrepresented in the both the Zymo and ATCC environmental mock communities. While sequencing bias for *Salmonella* could not be accounted for in the ATCC community because it is not included in the ATCC non‐MDA sequencing control, comparisons of the theoretical expected relative abundances of *Salmonella* to the relative abundances of *Salmonella* in the ATCC environmental mock samples following MDA all showed decreased relative abundances in the MDA samples regardless of spike‐in amounts. In the highest percent spike‐in *Salmonella* sample (0.0001 ng community DNA spiked with 1.8 × 10^3^ *Salmonella* genome copies) with an expected theoretical relative abundance of 90.9% *Salmonella* in the community, *Salmonella* only comprised of 63.8% of the final MDA community. For all samples and inputs in both the Zymo and ATCC environmental mock communities, *Salmonella* showed a mean of 72.4% ± 2.7% reduction from the expected total abundance of *Salmonella* after MDA. In the Zymo community, 14.1% of this bias could be attributed to sequencing based on the non‐reference sequencing control.

Evaluating reads mapped to multiple genomes (multi‐hit) with BLAST queries did not impact the taxa relative abundance biases observed in the above community results. See SM for further details under “Multi‐Hit Read Mapping” (Figure A5a–c).

#### 3.3.4 *Salmonella* Sequencing Performance Metrics Following Mda

**Figure 3:**
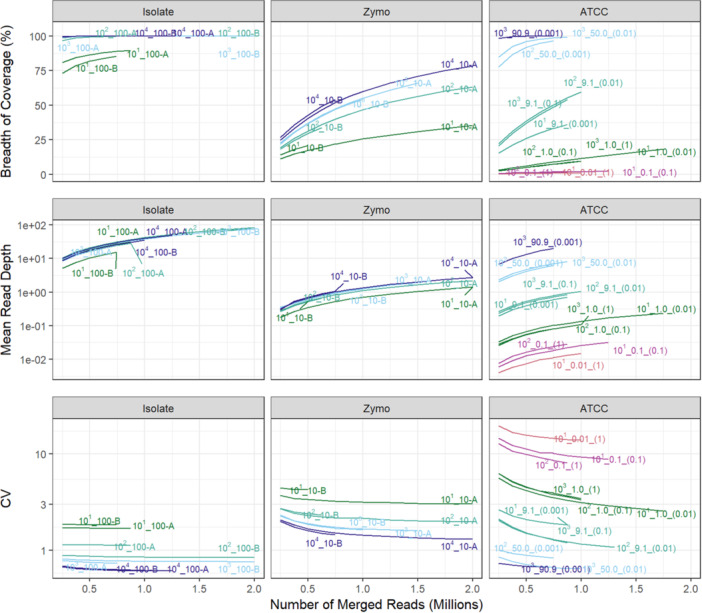
Performance metrics of *Salmonella* spp. genome in MDA communities at varying sequencing depths. Breadth of coverage, mean read depth and uniformity of coverage (CV) of *Salmonella* spp. in MDA isolate and mixed‐microbial mock communities based on reads mapped to the *Salmonella* LT2 (Isolate and ATCC) or FDAARGOS (Zymo) reference genome. Sample names for the isolate and Zymo community correspond to the log10 order of the *Salmonella* genome copy number and the percent of *Salmonella* in the total MDA community inputs. Sample names for the (c) ATCC environmental community correspond to the log10 order of the *Salmonella* genome copy number, the percent of *Salmonella* in the total MDA community, and the initial mass (ng DNA) input of the ATCC environmental community before being spiked with the *Salmonella* isolate.

Sequencing performance on *Salmonella* in the isolate and mock communities was evaluated using a single‐genome mapping approach. In isolate samples, starting MDA inputs of 0.1 and 0.01 ng DNA resulted in > 96% *Salmonella* LT2 genome coverage and a mean read depth > 8 at a sequencing depth of 250,000 merged, paired end reads (Figure 3, Table A8). Increasing the sequencing depth to 500,000 reads for the same samples increased the breadth of coverage to > 99% and a mean read depth > 16. Isolate samples with starting inputs of 0.001 ng DNA (1.8 × 10^1^ genome copies) showed a much lower breadth of coverage (73% and 81% at 250,000 merged reads) compared to higher DNA input samples. At a sampling depth of 625,000 merged reads, breadth of coverage for starting inputs of 0.001 ng DNA for duplicates A and B increased to 88% and 85%, respectively. For all isolate samples, mapped reads and mean read depths increased on average 119,667 ± 116 reads and 4.5 ± 0.1 reads, respectively, for each additional 125,000 reads sequenced in the analysis.

**Figure 4:**
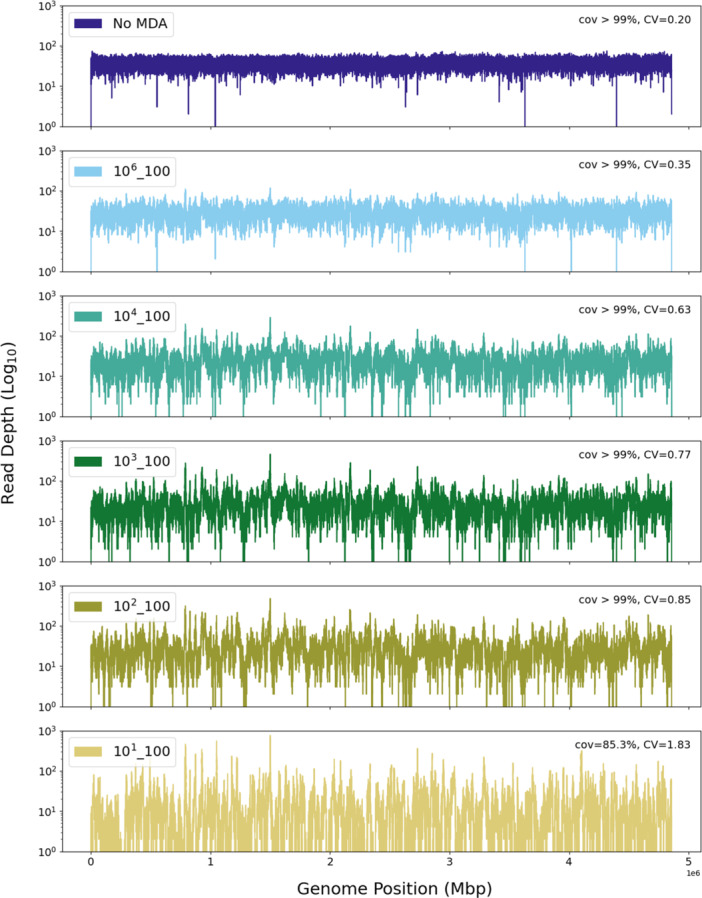
Effect of *Salmonella* LT2 input copy numbers on genome coverage following MDA in isolate community. Depth of coverage plots for 10^1^–10^4^ and 10^6^ *Salmonella* LT2 copy number inputs into MDA. A non‐MDA sequencing control (No MDA) is included for comparison. Coverage is based on mapped reads to the LT2 genome at a sequencing depth of 742,754 reads for each sample. Sample names indicate the log10 order of the *Salmonella* genome copy number and the percent of *Salmonella* in the total MDA inputs. Included in the sample legends are breadth of coverage (cov) and coverage uniformity (CV).

**Figure 5:**
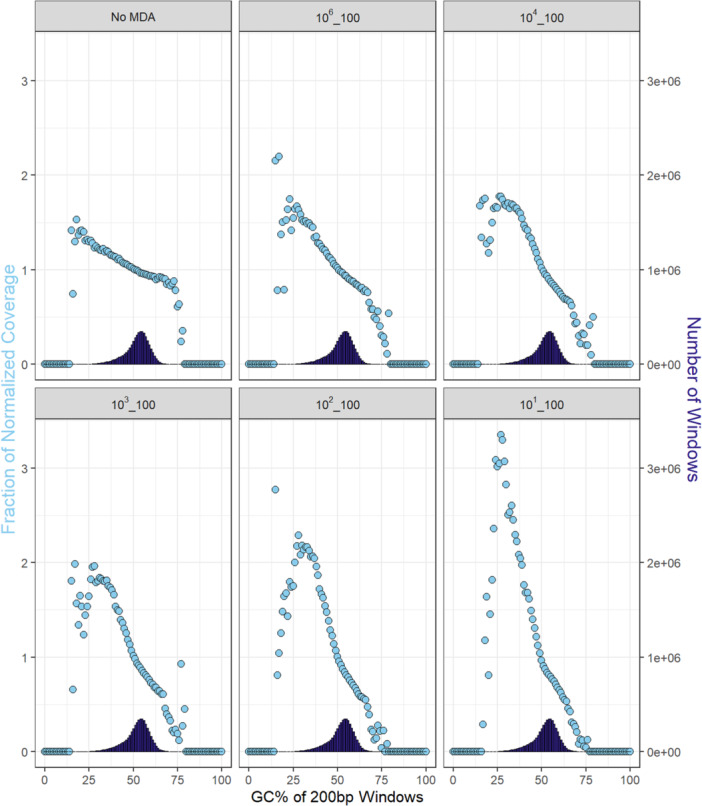
Effect of MDA for different starting input DNA amounts on %GC coverage. %GC bias curves based on output sequencing normalized coverage results for starting MDA inputs of 10^1^–10^4^ and 10^6^ copy numbers of *Salmonella* LT2 in the isolate WGA community samples. A non‐MDA sequencing control (No MDA) for the *Salmonella* LT2 isolate is shown for comparison. A 200‐bp sliding window was selected to determine the fraction of normalized coverage mapped to the *Salmonella* LT2 reference genome. A horizontal line at 1 for the fraction of normalized coverage (all 200 bp windows covered equally at all GC percentages) indicates no %GC bias; steeper curves indicate greater %GC bias. Sample names indicate the log10 order of the *Salmonella* genome copy number and the percent of *Salmonella* in the total MDA inputs.

Isolate samples with starting inputs of 0.1 ng DNA had the highest coverage uniformity with CV values < 0.62 at sequencing depths of 1 million merged reads. Coverage uniformity decreased with decreasing starting input amounts, but all CV values for the isolate samples remained below 1.85 even at sequencing depths as low as 250,000 reads. Coverage plots showed trends of increasing read depth, variation, and missing coverage across the genome following MDA with decreasing starting DNA input amounts (Figure 4). GC bias also increased with lower starting isolate DNA inputs as demonstrated by the increase in curve height and steepness shown in the GC bias plots (Figure 5).

In the Zymo mock community, 1.8%–2.9% of reads mapped to *Salmonella* FDAARGOS following MDA, with the highest input amount (1.0 ng DNA mock community) having the greatest percentage of mapped *Salmonella* reads (Figure 3, Table A8). Increasing the number of sequenced reads from 500,000 to 1,000,000 also increased the breadth of coverage of the *Salmonella* genome by ~1.5× and mean read depth by ~2.0%. At a sequencing depth ≥ 1,000,000 reads, > 50% of the *Salmonella* genome was covered at a mean read depth of > 1.2 for MDA community input amounts of 0.1 ng DNA and 1.0 ng DNA. Coverage uniformity (CV) for *Salmonella* at 500,000 reads ranged from 1.4 to 1.68 for starting MDA community inputs of 1.0–0.1 ng DNA but decreased (CV = 2.2–3.2) with smaller input amounts of 0.01–0.001 ng DNA. Zymo community coverage plots also showed trends of increasing read depth variation and missing coverage across the *Salmonella* genome with decreasing MDA input DNA amounts (Figure A6).

For the ATCC environmental mock community samples with *Salmonella* spiked into the sample, sequencing performance of *Salmonella* LT2 improved with increasing ratio of spike‐to‐community DNA inputs. In samples with ≥ 50% *Salmonella* LT2 in the MDA input community, breadth of spike‐in coverage was ≥ 90%, mean read depth was ≥ 4 reads, and CV was < 0.89 at a sequencing depth as low as 500,000 reads. For MDA inputs of 9.1% *Salmonella* DNA, breadth of coverage decreased to between 25.6% and 38.5%, mean depth decreased to < 1 read, and CV increased to 1.50–2.1 for 500,000 reads. MDA inputs of 0.1% *Salmonella* DNA performed poorly with a < 2.4% breadth of coverage, ≤ 0.03 mean read depth, and a CV > 8.0 at a sequencing depth of ~1.0 million reads. The ATCC environmental mock community coverage plots showed trends of increasing read depth variation and missing coverage across the genome as MDA starting *Salmonella* spike‐in amounts decreased and mock community input amounts decreased (Figure A7a,b).

## 4 Discussion

The ability to detect, and potentially characterize, pathogens by sequencing from environmental samples is critical for many public health applications, including outbreak response and prevention. In this study we used culture‐independent WGA with the Qiagen REPLI‐g MDA kit to evaluate and compare overall sequencing performance and detection sensitivity for *Salmonella* spp. in pure isolate samples and mock environmental microbial community samples at various concentrations.

In isolate MDA samples, *Salmonella* sequence recovery and identification performed well at MDA inputs as low as 0.001 ng DNA (1.8 × 10^2^ genome copies). Based on sequencing depths of as few as 500,000 merged 250 bp paired‐end reads, > 99% of the *Salmonella* genome was covered at > 16X coverage for MDA starting DNA inputs of 0.001–0.1 ng. At the lowest input of 0.0001 ng DNA, however, *Salmonella* showed a moderate decrease in amplification performance with a 13%–15% reduction in genome breadth of coverage and a decrease in coverage uniformity (1.1–1.2 increase in CV values). These data suggest that at MDA inputs of ≤ 0.001 ng, the effects of stochastic amplification and amplification biases become more pronounced and may impact *Salmonella* genome detection. MDA relies on the isothermal extension of random hexamer primers and exponential amplification of multi‐branched structures; thus, the lower the MDA input DNA amount, the greater the effect of under‐ or over‐amplification of genomic regions (Huang et al. 2015). Furthermore, with lower MDA inputs, preferential amplification of laboratory and reagent contaminates can occur (Blainey and Quake 2011; Woyke et al. 2011; Zhang et al. 2006), which was shown in this study by increased reads mapped to *E. coli*, vectors, and Φ29, as well as a reduction in the percentage of reads mapped to the *Salmonella* genome.

In our mixed‐microbial communities (Zymo and ATCC), *Salmonella* was consistently underrepresented following MDA as measured by the percent relative abundance of *Salmonella*‐identified reads compared to the expected composition. Across both community samples, *Salmonella* showed 72.4% ± 2.7% mean reduction from the expected total abundance. Genome abundance distortion was also observed across multiple taxa following MDA. Previous studies on microbial communities have also shown significant alterations and discordance of taxa abundances following MDA (Abulencia et al. 2006; Ahsanuddin et al. 2017; Direito et al. 2014; Probst et al. 2015). The qualitative patterns of abundance distortion in our study remained consistent and predictable among *Salmonella* and the community taxa indicating that the driving force of these biases is likely not stochastic amplification. This pattern of amplification bias was also seen in isolate MDA samples, where even at high DNA inputs of 10.0 ng there were areas of localized amplification bias as indicated by peaks and troughs in coverage plots. These biases, however, became more pronounced as the MDA inputs decreased, indicating that amplification bias at these locations intensified at low template levels. Ahsanuddin et al. (2017) found a similar pattern of increased localized enrichment in the *M. luteus* genome when using lower levels of DNA input with the Qiagen REPLI‐g MDA method.

WGA amplification bias has been noted to occur in GC‐rich or GC‐poor genome regions (Ahsanuddin et al. 2017; Sabina and Leamon 2015). This may occur because during strand displacement and primer extension in DNA replication, Phi29 undergoes more frequent pause states in GC‐rich DNA segments, contributing to uneven amplification among the taxa genomes (Morin et al. 2012). In the isolate samples, MDA amplification bias related to percent GC was evidenced by the isolate percent GC bias curves. Amplification bias was more pronounced for lower MDA inputs of the isolate samples, as demonstrated by the steep increase in the percent GC‐bias curves with decreasing amounts of DNA input. While genome GC content alone could not account for the distortion in genome amplification among taxa following MDA in the mixed‐microbial communities, there was evidence of percent GC bias among the taxa. For example, GC‐rich genomes *P. aeruginosa* (Zymo, GC = 66.2%) and *P. fluorescens* (ATCC, GC = 60%) both exhibited a large decrease in relative abundance compared to non‐MDA sequencing controls in their respective communities, while relatively GC‐poor genomes *S. aureus* (Zymo, GC = 32.9%) and *S. epidermis* (ATCC, GC = 32.1%) were consistently overrepresented in relative abundance in the MDA samples. Together these findings indicate that genome GC content likely had an important role in the amplification biases seen in this study, but there may be other factors driving distortion of community composition that were not evaluated in this study, such as DNA integrity of individual genomes or secondary physical structures in the genomes that could impact MDA efficiency among different taxa. For example, degraded (Dean et al. 2002) or fragmentated DNA (Direito et al. 2014) has also been shown to distort MDA amplification by reducing DNA accessibility for random primer extension and ensuing exponential amplification.

Despite potential for amplification bias as shown in this study, MDA may still provide a useful, culture‐independent tool for amplifying DNA of low abundance targets in environmental samples and may improve the ability to detect pathogens, like *Salmonella*, in complex matrices. Even with DNA inputs as low as 0.0001 ng, all samples in this study had MDA outputs of ≥ 584 ng/µL (total DNA amounts ≥ 29.2 µg), and ≥ 83.6% of sequencing reads mapped uniquely to expected genomes. This study demonstrates a tool for improving output from metagenomic and sequence‐based laboratory methods that may not otherwise be practical in low biomass or low target environmental samples. While some relative abundance distortion of microbial communities was evident in metagenomic sequencing following MDA that may limit the utility of this method, other applications related to the identification and detection of pathogens can benefit from MDA. Such applications include outbreak response, where high‐resolution genomic matching of pathogens in the environment to clinical cases is necessary to identify exposure routes and prevent future outbreaks, as well as environmental epidemiology where pathogen relatedness in environmental samples can aid in understanding changes in health risks from environmental exposure.

To this end, the *Salmonella‐*specific *ttr* gene‐based detect assay (Hopkins et al. 2009; Malorny et al. 2004) has been widely used for determining *Salmonella* spp. contamination in different environments. In the current study, with the exception of one sample that remained below the limit of detection (LOD = 0.1% *Salmonella* DNA in ≥ 0.1 ng DNA mixed community), WGA resulted in a 9.6 × 10^3^–1.1 × 10^7^ ‐fold increase in estimated *ttr* copy numbers in mock environmental community samples without the need for time consuming, culture‐based amplification. Furthermore, this estimated improvement in *Salmonella* detection sensitivity following MDA may be conservative, because for seven out of eight isolate samples, the read depth for genome positions covered by the *ttr* gene was between 1.5‐ to 6.1‐fold less than the mean genome coverage based on metagenomic shotgun sequencing results.

## 5 Conclusion

Molecular assays which use targeted amplification for detection and genomic characterization of pathogenic microbes may benefit from MDA of low biomass, mixed microbial samples. However, microbial detection and genomic characterization using untargeted metagenomic methods following MDA may be unreliable and require further development. Furthermore, additional consideration must be taken when investigating environmental samples, including confounding factors apart from MDA itself that affect metagenomic community analyses; these may include potential polymerase inhibitors (Tsai and Olson 1992; Wilson 1997) present in the environmental matrix of interest and genome recovery biases in DNA extraction, such as preferential lysis of Gram–taxa (Brooks et al. 2015; Guo and Zhang 2013; Shehadul Islam et al. 2017). While this study demonstrated limitations of MDA associated with amplification biases and amplification of reagent contamination, use of new technologies, such microfluidics via microchannel MDA (Li et al. 2017) and random, primer‐free WGA (Picher et al. 2016) may help overcome these limitations and advance WGA technology applications for improved pathogen detection in environmental samples.

## Author Contributions

**Ann Arfken:** investigation (lead), formal analysis (lead), methodology (equal), data curation (lead), project administration (lead), validation (lead), conceptualization (supporting), visualization (lead), writing – original draft (lead), writing – review and editing (equal). **Jeffrey Mercante:** conceptualization (supporting), writing – review and editing (equal). **Mia Mattioli:** conceptualization (lead), methodology (equal), project administration (supporting), review and editing (equal).

## Funding

The authors received no specific funding for this work.

## Ethics Statement

The authors have nothing to report.

## Conflicts of Interest

The authors declare no conflicts of interest.

## Supporting information


**Figure A1:** Composition of MDA Controls. **Figure A2:** Genome coverage of *E. coli* genome in negative template controls following MDA. **Figure A3:** Effect of *Salmonella* LT2 input copy numbers on genome coverage following MDA in isolate community (Duplicate Samples, Isolate‐A). **Figure A4:** Effect of *Salmonella* input copy numbers on genome coverage following MDA in the Zymo mixed‐microbial mock community (Duplicate Samples, Zymo‐B). **Figure A5:** BLAST analysis of multi hit reads from MDA Fastq‐Screen results. **Figure A6:** Effect of *Salmonella* input copy numbers on genome coverage following MDA in the Zymo mixed‐microbial mock community. Depth of coverage plots for 10^1^‐10^4^ *Salmonella* FDAARGOS copy number inputs into MDA. **Figure A7:** Effect of *Salmonella* input copy numbers and different ratios on genome coverage in ATCC environmental mixed‐microbial community following MDA. **Table A1:** Composition of commercial DNA mixed‐microbial mock communities. **Table A2:** Relative abundances of genomes in (a) NTC and (b) positive REPLI‐g controls are based on a multi‐map read analysis using Fastq‐Screen and customized databases for each isolate or mixed‐microbial mock community following MDA. **Table A3:** Mapping statistics to *E. coli* genome for REPLI‐g NTCs following MDA using a single‐mapping approach with Bowtie2. **Table A4:** Starting input DNA amounts and final output DNA concentration and amounts following MDA for positive and negative controls. **Table A5:** Quantification results of the *Salmonella ttr* gene in isolate and mock community samples following MDA. **Table A6:** Mean read depth (total genome) compared to mean read depth covering the *ttr* gene (1,469,333 – 1,469,427 bp) in MDA sequencing results for *Salmonella* isolate based on mapping results to the *Salmonella* LT2 reference genome. **Table A7:** Illumina MiSeq sequencing results following QC. **Table A8:** *Salmonella* spp. genome performance results following MDA.

## Data Availability

Sequencing data from this study are available through NCBI (National Center for Biotechnology Information) under Bioproject PRJNA994911, accession numbers SAMN36452344‐36452375. Custom codes and scripts used for bioinformatic analyses are accessible at https://github.com/CDCgov/WDPB_EMEL/tree/main/manuscripts/WGA_Evaluation.
