# Supplementary material for: Improved Sensitivity of Quantitative Polymerase Chain Reaction and Next Generation Sequencing for Detection of Salmonella spp. in Mixed Environmental Communities Using Whole Genome Amplification
Source: Microbiologyopen. 2025 Dec 12;14(6):e70194. doi: 10.1002/mbo3.70194 (PMC12699375; doi:10.1002/mbo3.70194)
Supplement: Supplementary file 1 — Figure A1: Composition of MDA Controls. Figure A2: Genome coverage of E. coli genome in negative template controls following MDA. Figure A3: Effect of Salmonella LT2 input copy numbers on genome coverage following MDA in isolate community (Duplicate Samples, Isolate‐A). Figure A4: Effect of Salmonella input copy numbers on genome coverage following MDA in the Zymo mixed‐microbial mock community (Duplicate Samples, Zymo‐B). Figure A5: BLAST analysis of multi hit reads from MDA Fastq‐Screen results. Figure A6: Effect of Salmonella input copy numbers on genome coverage following MDA in the Zymo mixed‐microbial mock community. Depth of coverage plots for 101‐104 Salmonella FDAARGOS copy number inputs into MDA. Figure A7: Effect of Salmonella input copy numbers and different ratios on genome coverage in ATCC environmental mixed‐microbial community following MDA. Table A1: Composition of commercial DNA mixed‐microbial mock communities. Table A2: Relative abundances of genomes in (a) NTC and (b) positive REPLI‐g controls are based on a multi‐map read analysis using Fastq‐Screen and customized databases for each isolate or mixed‐microbial mock community following MDA. Table A3: Mapping statistics to E. coli genome for REPLI‐g NTCs following MDA using a single‐mapping approach with Bowtie2. Table A4: Starting input DNA amounts and final output DNA concentration and amounts following MDA for positive and negative controls. Table A5: Quantification results of the Salmonella ttr gene in isolate and mock community samples following MDA. Table A6: Mean read depth (total genome) compared to mean read depth covering the ttr gene (1,469,333 – 1,469,427 bp) in MDA sequencing results for Salmonella isolate based on mapping results to the Salmonella LT2 reference genome. Table A7: Illumina MiSeq sequencing results following QC. Table A8: Salmonella spp. genome performance results following MDA. [file MBO3-14-e70194-s001.docx]

Appendices

Control Composition

Sequences identified in the REPLI-g negative template controls for all communities using FastQ-Screen were *E. coli*, vectors, and Φ29, as well as reads that multi-mapped to more than one genome (Fig. A1a, Table A2a). Non-identified reads comprised ≤ 0.2% relative abundance of the mapped reads. Reads mapping exclusively to *E. coli*, vectors, Φ29 were not found in the REPLI-g positive controls (Fig.A1b, Table A2b).

The mean number of REPLI-g negative control reads mapped to the *E. coli* reference (GCF_000005845.2) for all community samples was 2.5 x 10^5^ ± 2.7 x 10^4^ reads, with a mean mapped read relative abundance of 35.5 ± 6.1%, a mean breadth of coverage of 0.6 ± 0.1%, and a mean CV of 31.5 ± 1.6 (Table A3). Depth of coverage plots for each of the REPLI-g negative template samples showed mostly inconsistent and uneven coverage of mapped reads across the *E. coli* genome (Fig. A2). In the REPLI-g positive controls, ≥ 99.8% of reads correctly mapped to the human reference (GCF_000001405.39) for the isolate samples (Fig. A1b, Table A3b). In mixed-microbial community samples, >90.6% of reads correctly mapped to the *Salmonella* LT2 genome, with the remaining reads multi-mapping to more than one genome in their respective communities.

Multi-hit Read Mapping

Reads mapping to more than one reference genome (multi-hit) made up a mean relative abundance of 7.6 ± 0.3% across all mixed-microbial community and isolate samples following MDA (Fig. A5a-c). Among all samples, on average 35.4 ± 4.1% of reads designated as multi-hits did not have a BLAST match (no-hit) of at least 90% coverage and 90% percent similarity to any genome in the sample’s respective reference genome collection. This may indicate possible chimera or ambiguous regions within the reads. Reads multi-mapping to both vectors and *Staphylococcus* spp. were the next most abundant multi-hit reads after ‘no hits’ in the Zymo and ATCC environmental mock community samples following MDA, with a mean of 26.3 ± 2.5% relative abundance (Figs. A5b-c).

Tables and Figures

**Tables**

**Table A1.** Composition of commercial DNA mixed-microbial mock communities.

**Table A2.** Relative abundances of genomes in (a) NTC and (b) positive REPLI-g controls are based on a multi-map read analysis using Fastq-Screen and customized databases for each isolate or mixed-microbial mock community following MDA. A & B in sample name indicate duplicates.

**Table A3.** Mapping statistics to *E. coli* genome for REPLI-g NTCs following MDA using a single-mapping approach with Bowtie2. A & B in sample name indicate duplicates.

**Table A4.** Starting input DNA amounts and final output DNA concentration and amounts following MDA for positive and negative controls.

**Table A5.** Quantification results of the *Salmonella* *ttr* gene in isolate and mock community samples following MDA. B.D. denotes below detection limit. Sample names for the isolate and Zymo community correspond to the log10 order of the *Salmonella* genome copy number and the percent of *Salmonella* in the total MDA community inputs; sample names for the ATCC environmental community correspond to the log10 order of the *Salmonella* genome copy number, the percent of *Salmonella* in the total MDA community, the initial mass (ng DNA) input of the ATCC environmental community prior to being spiked with the *Salmonella* isolate.

**Table A6.** Mean read depth (total genome) compared to mean read depth covering the *ttr* gene (1,469,333 – 1,469,427 bp) in MDA sequencing results for *Salmonella* isolate based on mapping results to the *Salmonella* LT2 reference genome. Sample name corresponds to the log10 order of the Salmonella genome copy number and the percent of *Salmonella* in the total MDA community inputs; A and B indicate duplicates.

**Table A7.** Illumina MiSeq sequencing results following QC. Sample names for the isolate and Zymo community correspond to the log10 order of the *Salmonella* genome copy number and the percent of *Salmonella* in the total MDA community inputs; A & B indicate duplicates. Sample names for the ATCC environmental community correspond to the log10 order of the *Salmonella* genome copy number, the percent of *Salmonella* in the total MDA community, and the initial mass (ng DNA) input of the ATCC environmental community prior to being spiked with the *Salmonella* isolate.

**Table A8.** *Salmonella* spp. genome performance results following MDA. Sample names for the isolate and Zymo community correspond to the log10 order of the *Salmonella* genome copy number and the percent of *Salmonella* in the total MDA community inputs; A & B indicate duplicates. Sample names for the ATCC environmental community correspond to the log10 order of the *Salmonella* genome copy number, the percent of *Salmonella* in the total MDA community, and the initial mass (ng DNA) input of the ATCC environmental community prior to being spiked with the *Salmonella* isolate.

**Figures**


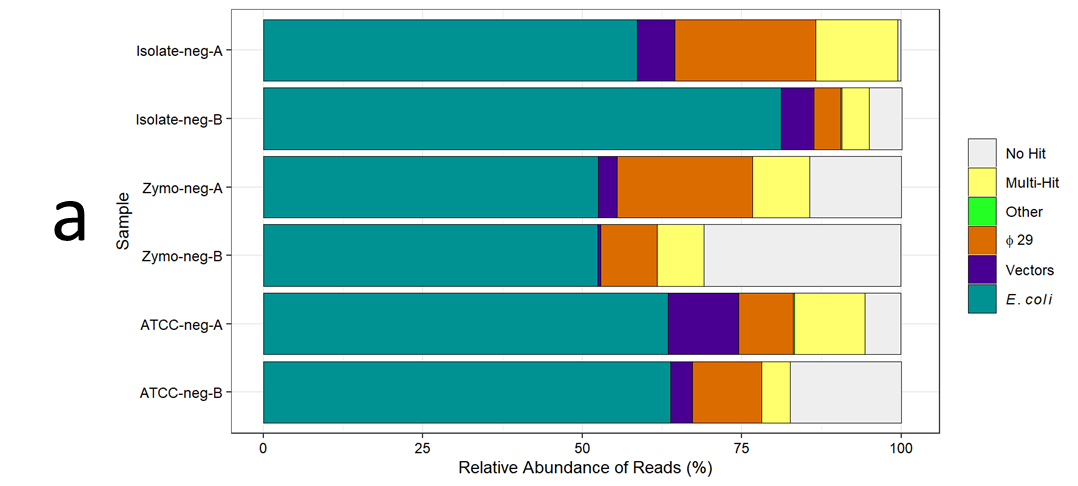


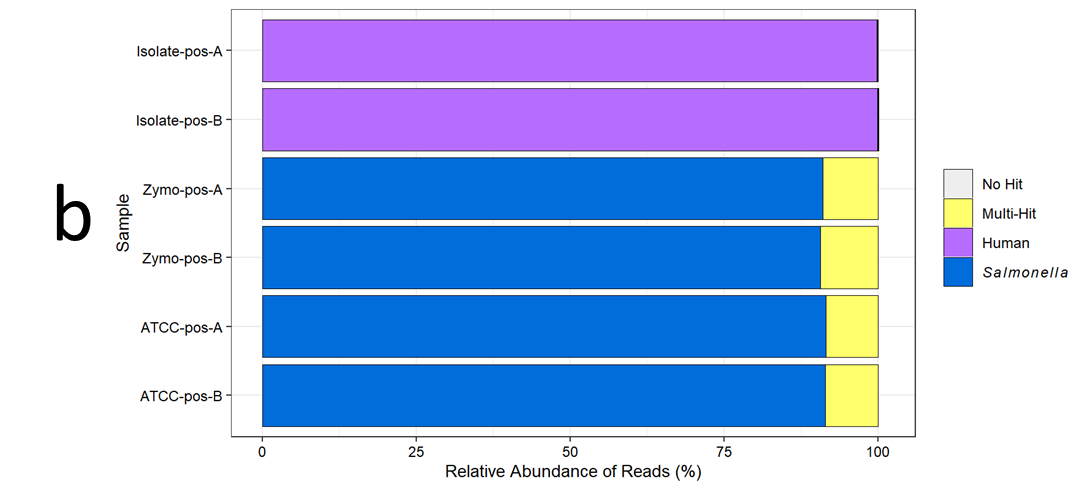


**Figure A1. Composition of MDA Controls.** Relative abundances of taxa based on a multi-map read analysis using Fastq-Screen for (a) NTCs and (b) positive controls following MDA. Each multi-map analysis was run with the appropriate customized community reference database. Sample names indicate the corresponding experimental community. 10 ng of *Salmonella* LT2 and human genomic DNA served as positive controls for the mixed (ATCC and Zymo) and isolate communities, respectively.


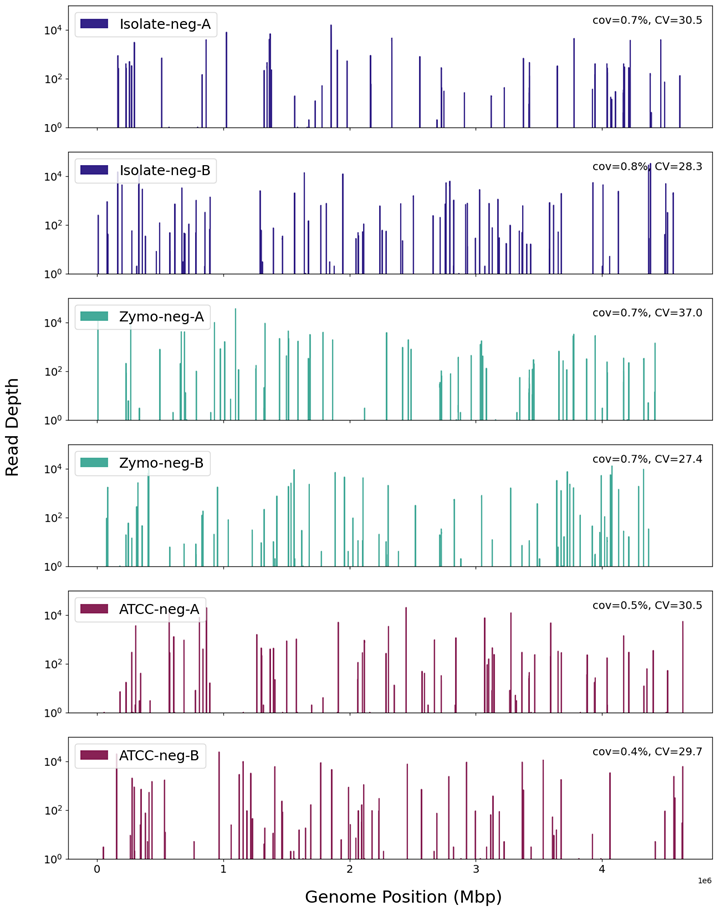


**Figure A2. Genome coverage of *E. coli* genome in negative template controls following MDA.** WGA/WMA negative template control sample reads mapped to the *E. coli* genome at a sequencing depth of 742,754 reads. Sample names for the negative controls correspond to the appropriate MDA community. Included in the sample legend are breadth of coverage (cov) and coverage uniformity (CV).

**
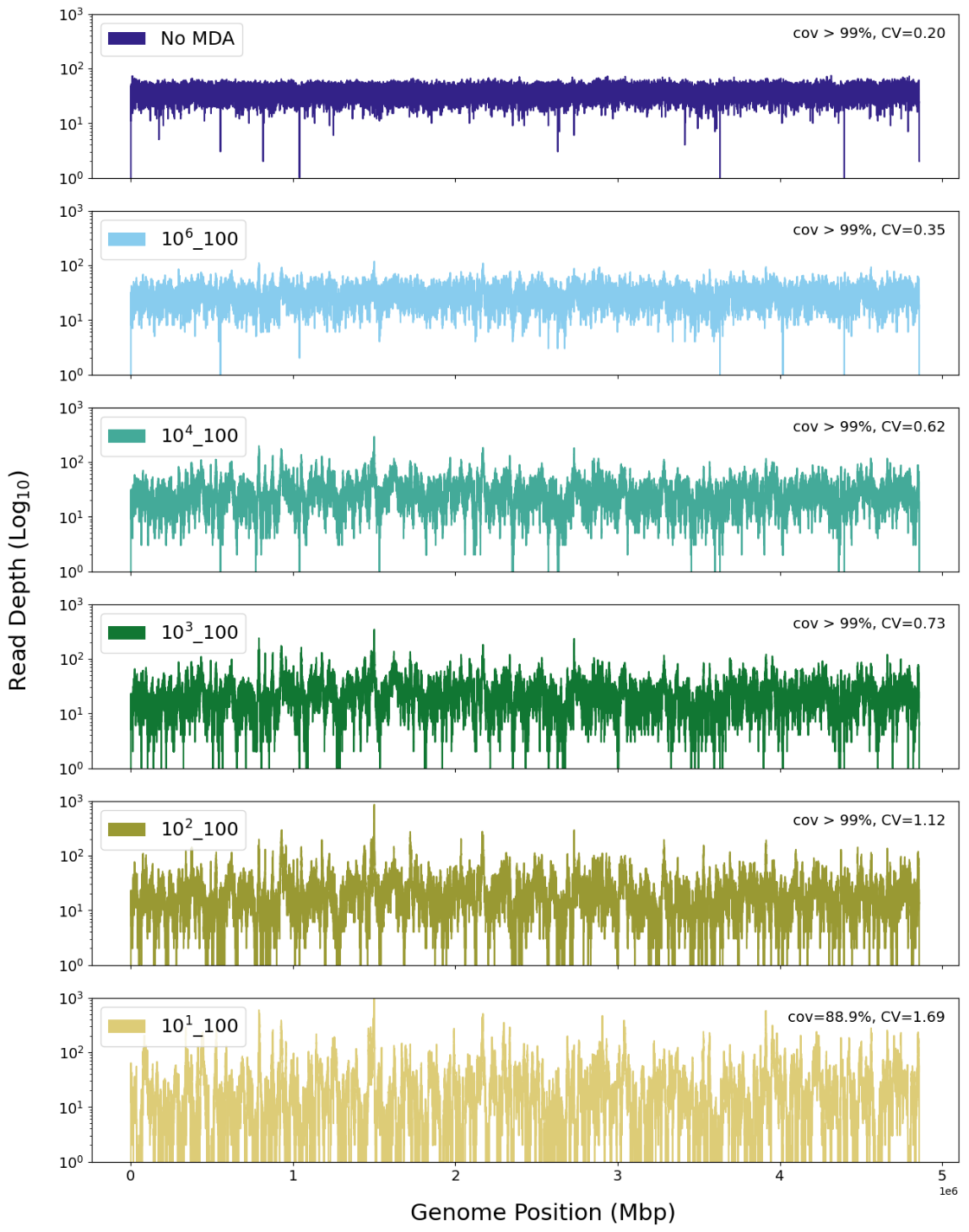
**

**Figure A3.** **Effect of *Salmonella* LT2 input copy numbers on genome coverage following MDA in isolate community (Duplicate Samples, Isolate-A).** Depth of coverage plots for 10^1^-10^4^ and 10^6^ *Salmonella* LT2 copy number inputs into MDA. A non-MDA reference sequencing control (No MDA) is included for comparison. Coverage is based on mapped reads to the LT2 genome at a sequencing depth of 742,754 reads for each sample. Sample names indicate the log10 order of the *Salmonella* genome copy number and the percent of *Salmonella* in the total MDA inputs. Included in the sample legends are breadth of coverage (cov) and coverage uniformity (CV).


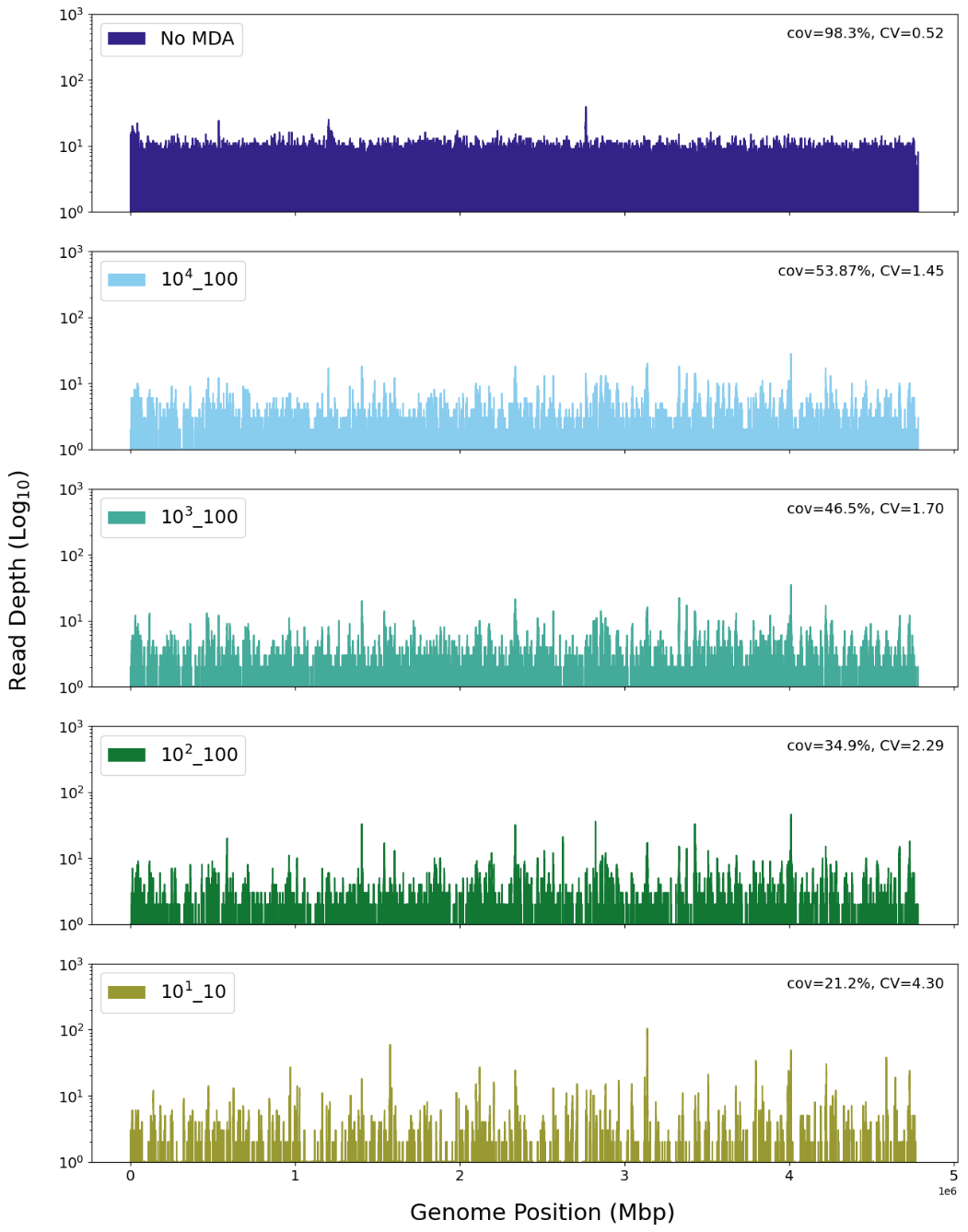


**Figure A4.** **Effect of *Salmonella* input copy numbers on genome coverage following MDA in the Zymo mixed-microbial mock community (Duplicate Samples, Zymo-B).** Depth of coverage plots for 10^1^-10^4^ *Salmonella* FDAARGOS copy number inputs into MDA. A non-MDA reference sequencing control (No MDA) is included for comparison. Coverage is based on mapped reads to the FDAARGOS genome at a sequencing depth of 742,754 reads for each sample. Sample names correspond to the log10 order of the *Salmonella* genome copy number and the percent of *Salmonella* in the total MDA community inputs. Included in the sample legend are breadth of coverage (cov) and coverage uniformity (CV).


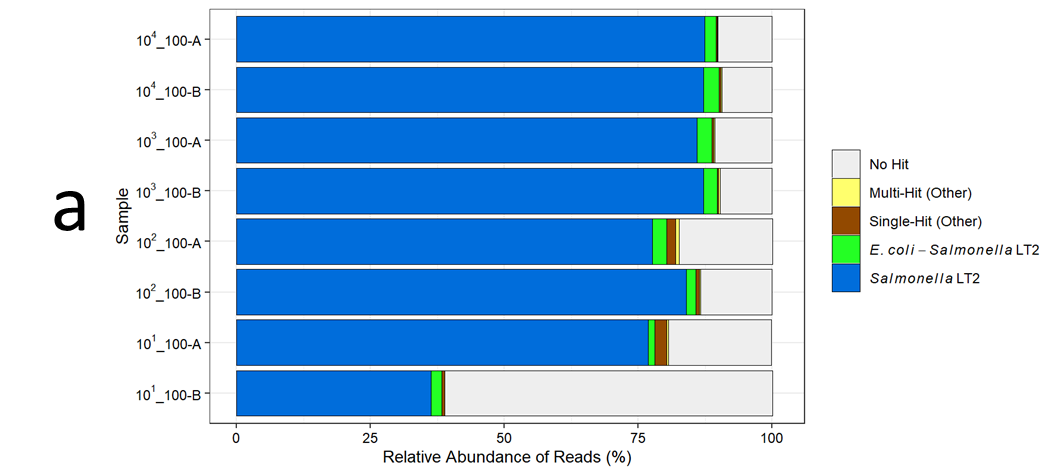


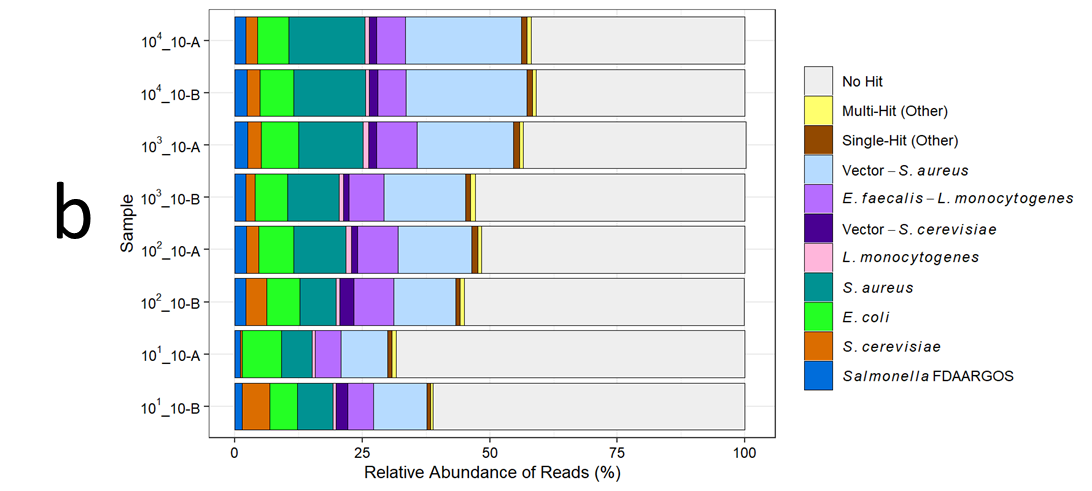


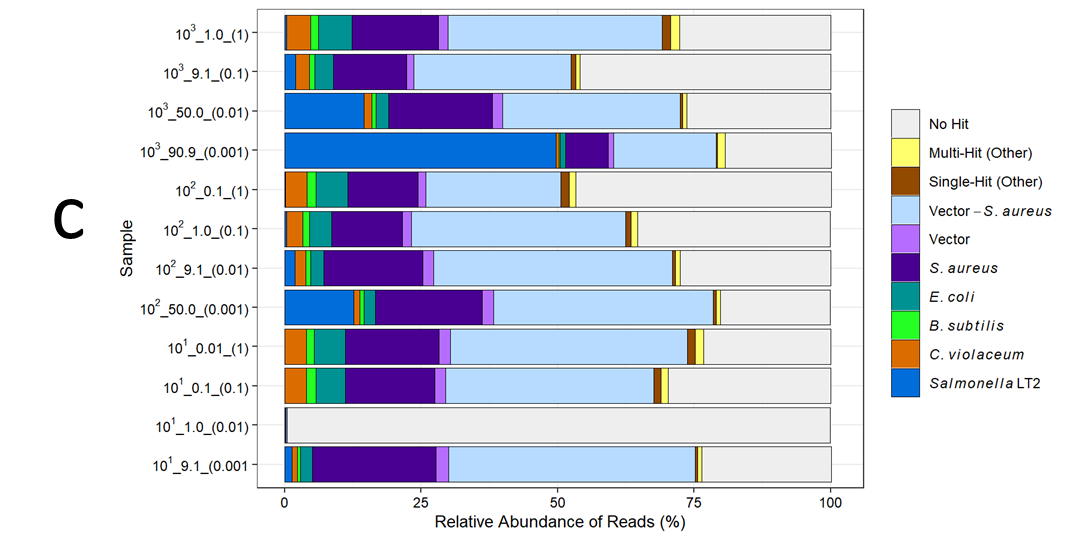


**Figure A5. BLAST analysis of multi hit reads from MDA Fastq-Screen results.** Taxonomic identification of reads mapping to more than one reference genome (Multi-Hit) for (a) isolate (100% *Salmonella*) (b) Zymo (10% *Salmonella*) and (c) ATCC environmental (mixed % *Salmonella*) MDA communities using BLAST. BLAST alignments were performed with extracted Fastq-Screen against the appropriate customized community reference database. Taxa identification is based on top hit bit scores. Unique top hits are represented by a single taxon and tied top hits are represented by multiple taxa in the legend. Relative abundances are based on total numbers of extracted multi hit reads. Sample names for the (a) isolate and (b) Zymo community correspond to the log10 order of the *Salmonella* genome copy number and the percent of *Salmonella* in the total MDA community inputs. Sample names for the (c) ATCC environmental community correspond to the log10 order of the *Salmonella* genome copy number, the percent of *Salmonella* in the total MDA community, and the initial mass (ng DNA) input of the ATCC environmental community prior to being spiked with the *Salmonella* isolate.

**
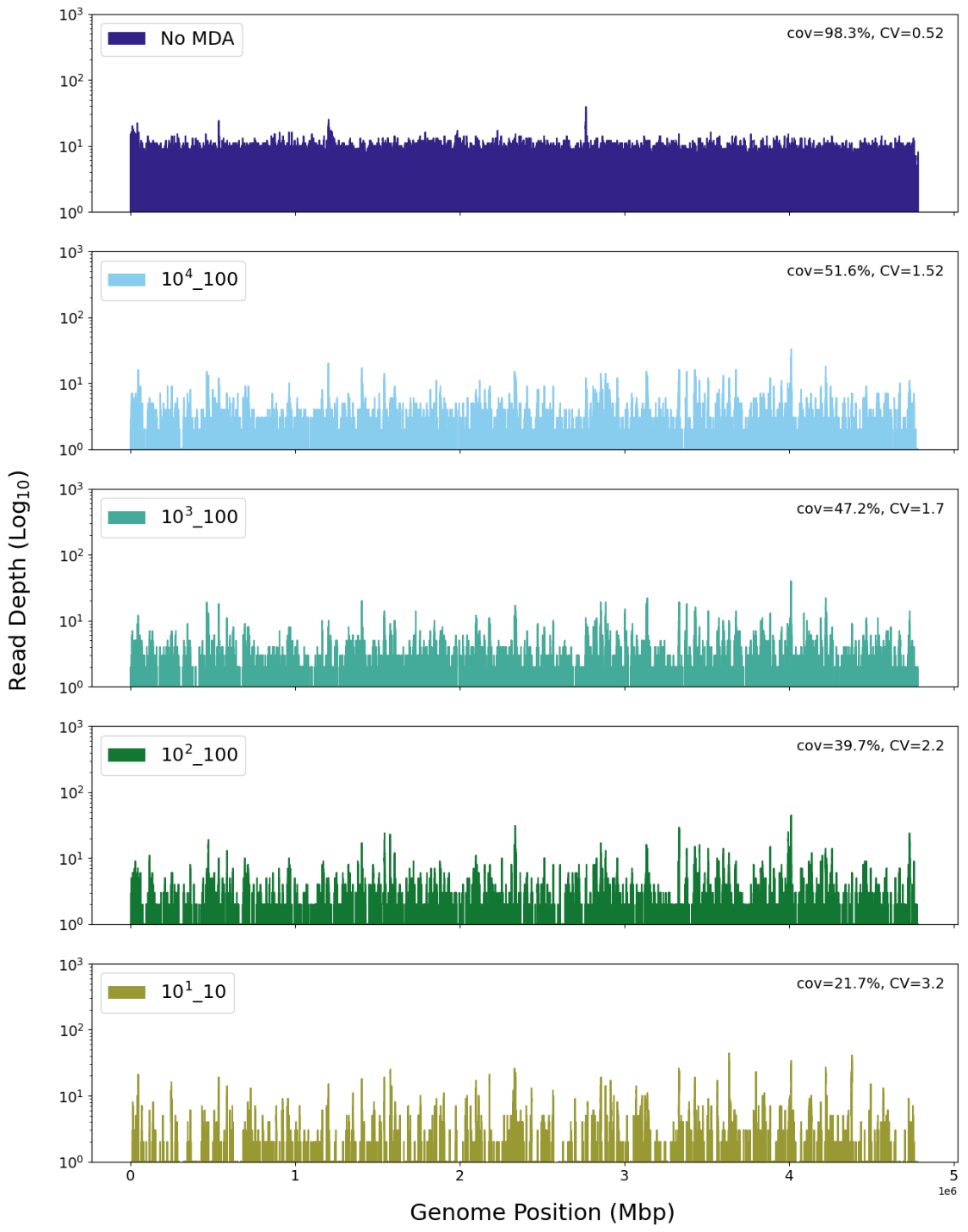
**

**Figure A6. Effect of *Salmonella* input copy numbers on genome coverage following MDA in the Zymo mixed-microbial mock community.** Depth of coverage plots for 10^1^-10^4^ *Salmonella* FDAARGOS copy number inputs into MDA. A non-MDA reference sequencing control (No MDA) is included for comparison. Coverage is based on mapped reads to the FDAARGOS genome at a sequencing depth of 742,754 reads for each sample. Sample names correspond to the log10 order of the *Salmonella* genome copy number and the percent of *Salmonella* in the total MDA community inputs. Included in the sample legend are breadth of coverage (cov) and coverage uniformity (CV).


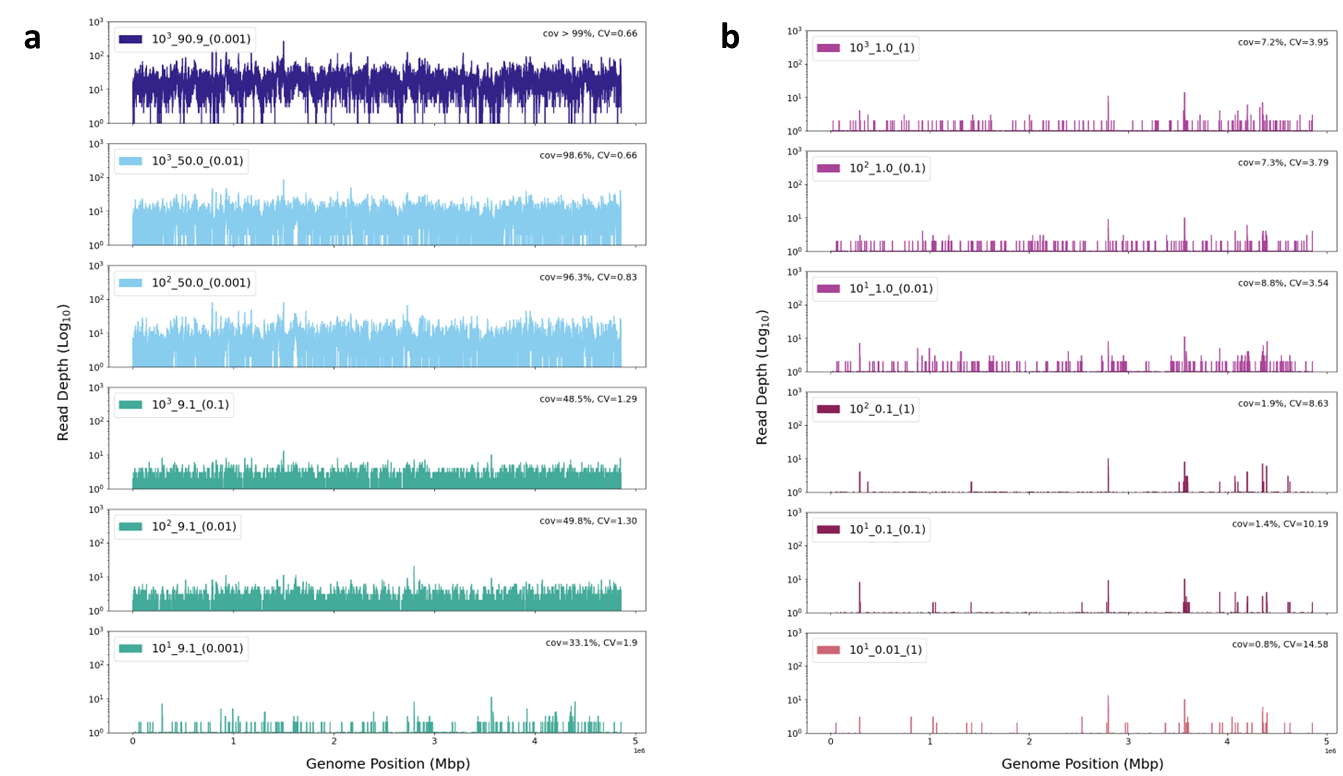


**Figure A7. Effect of *Salmonella* input copy numbers and different ratios on genome coverage in ATCC environmental mixed-microbial community following MDA.** Depth of coverage plots for (a) high percent *Salmonella* (9.1-90.9%) and (b) low percent *Salmonella* (0.01-1.0%) in starting input ATCC community input at different input genome copy numbers (10^1^-10^3^). Coverage is based on mapped reads to the LT2 genome at a sequencing depth of 742,754 reads for each sample. Sample names correspond to the log10 order of the *Salmonella* genome copy number and the percent of *Salmonella* in the total MDA community inputs. Included in the sample legend are breadth of coverage (cov) and coverage uniformity (CV).
